# Blood lactate in a cohort of patients with severe penetrating trauma at the intensive care unit in a university hospital

**DOI:** 10.1186/2197-425X-3-S1-A376

**Published:** 2015-10-01

**Authors:** M Gonzalez, J Ruiz, F Jaimes

**Affiliations:** Universidad de Antioquia, Medellin, Colombia; Internal Medicine, Universidad de Antioquia, Medellin, Colombia

## Introduction

The usefulness of the initial serum lactate as a prognostic marker in different critical conditions has been controversial. While some consider that an initial high lactate is enough to indicate higher mortality, others consider that the really important issue is lactate clearance. Specifically in penetrating trauma, there are no studies on this matter.

## Objectives

To estimate the effect of the initial values of lactate in hospital mortality of patients with severe penetrating trauma.

## Methods

In a retrospective cohort from October 2013 to October 2014 we included patients over 14 years old, admitted by the emergency service with surgical penetrating trauma and attended in the intensive care unit. At least one measurement of lactate at recruitment was required, as well as information on the mechanism of trauma, injured body area, revised trauma score (RTS), APACHE II, length of stay and mortality. The relationship between lactate levels and mortality was established by means of a logistic regression model.

## Results

Ninety five patients entered the institution with penetrating trauma and were subjected to surgery. However, 17 were not included in the study since they were not taken to the ICU or did not have a lactate test within the first 24 hours. Among 78 patients studied ninety six percent (n = 75) were men and the mean age was 29.5 years (SD = 11.4). The most common mechanism of trauma was a bladed weapon in 77% of the cases; 51% were exclusively wound of thorax and a 20.8% had a chest wound combined with other body area. The mean lactate level was 39.5 mg/dL on those alive and 97.6 mg/dL on the dead. The mean RTS was 5.2 and 14.1% of patients had a RTS less than 3. The APACHE II mean was 15.7 with ranges between 5 and 35. The mean hospital stay was 12.5 days and the hospital mortality rate was 10% (n = 8). The logistic regression model identified a linear relationship between the values of lactate and the risk of death, which remained after adjusting for the values of APACHE II (OR = 1.025; CI 95% = 1.001 - 1.05).

## Conclusions

The initial lactic acid level is confirmed as an independent risk factor for mortality in severe penetrating trauma in patients with postoperative management at ICU. The additional role of lactate clearance in the early hours must be defined; and the applicability of these findings depends on their replication in other institutions.

## Grant Acknowledgment

Universidad de Antioquia and Hospital Universitario San Vicente Fundacion
